# Postnatal PPARδ Activation and Myostatin Inhibition Exert Distinct yet Complimentary Effects on the Metabolic Profile of Obese Insulin-Resistant Mice

**DOI:** 10.1371/journal.pone.0011307

**Published:** 2010-06-25

**Authors:** Barbara L. Bernardo, Timothy S. Wachtmann, Patricia G. Cosgrove, Max Kuhn, Alan C. Opsahl, Kyle M. Judkins, Thomas B. Freeman, John R. Hadcock, Nathan K. LeBrasseur

**Affiliations:** 1 Cardiovascular, Metabolic and Endocrine Diseases, Pfizer Global Research and Development, Groton, Connecticut, United States of America; 2 Preclinical Statistics, Pfizer Global Research and Development, Groton, Connecticut, United States of America; 3 Investigative Pathology Laboratory, Pfizer Global Research and Development, Groton, Connecticut, United States of America; University of Tor Vergata, Italy

## Abstract

**Background:**

Interventions for T2DM have in part aimed to mimic exercise. Here, we have compared the independent and combined effects of a PPARδ agonist and endurance training mimetic (GW501516) and a myostatin antibody and resistance training mimetic (PF-879) on metabolic and performance outcomes in obese insulin resistant mice.

**Methodology/Principal Findings:**

Male ob/ob mice were treated for 6 weeks with vehicle, GW501516, PF-879, or GW501516 in combination with PF-879. The effects of the interventions on body composition, glucose homeostasis, glucose tolerance, energy expenditure, exercise capacity and metabolic gene expression were compared at the end of study. GW501516 attenuated body weight and fat mass accumulation and increased the expression of genes of oxidative metabolism. In contrast, PF-879 increased body weight by driving muscle growth and altered the expression of genes involved in insulin signaling and glucose metabolism. Despite their differences, both interventions alone improved glucose homeostasis. Moreover, GW501516 more effectively improved serum lipids, and PF-879 uniquely increased energy expenditure, exercise capacity and adiponectin levels. When combined the robust effects of GW501516 and/or PF-879 on body weight, adiposity, muscle mass, glycemia, serum lipids, energy expenditure and exercise capacity were highly conserved.

**Conclusions/Significance:**

The data, for the first time, demonstrate postnatal inhibition of myostatin not only promotes gains in muscle mass similar to resistance training,but improves metabolic homeostasis. In several instances, these effects were either distinct from or complimentary to those of GW501516. The data further suggest that strategies to increase muscle mass, and not necessarily oxidative capacity, may effectively counter insulin resistance and T2DM.

## Introduction

Exercise training positively affects body composition, energy expenditure, glucose homeostasis and insulin requirements and thus, remains a cornerstone in the prevention and treatment of type 2 diabetes mellitus (T2DM). The beneficial effects of exercise have fueled an interest in the development of pharmacologic interventions that mimic aspects of it, hence the term, *exercise mimetics*. While several studies have supported the role of endurance training, relatively little is known with respect to resistance training, or even less so the utility of a resistance-training mimetic, for countering insulin resistance and T2DM.

Skeletal muscle is a dynamic system and exhibits remarkable metabolic adaptations to exercise. Endurance exercise enhances the activity and expression of key signaling intermediates, transcriptional co-activators, and transcription factors that orchestrate glucose transport and fatty acid utilization in skeletal muscle and drive the biogenesis of mitochondria (reviewed in [1]). In turn, modified expression of several of these molecules, including CaMK [2], AMPK [3], [4], PGC1α[5], [6] and β[7], and PPARδ [8], directly alters metabolic outcomes in mice including adiposity, glucose tolerance, oxidative capacity, and endurance (or resistance to fatigue). Moreover, recent work has demonstrated that pharmacologic activation of AMPK and PPARδ increases expression of oxidative genes in skeletal muscle and running capacity in mice in a manner that partially mimics or enhances endurance exercise training [10]. Collectively, these data and other reports have supported therapeutic strategies that increase the oxidative and endurance capacity of skeletal muscle for obesity and T2DM.

In contrast to endurance exercise, the metabolic benefits of modifying skeletal muscle in a resistance training manner are not widely recognized. A limited number of clinical studies have demonstrated increased muscle mass and improved glycemic control in patients with T2DM following a resistance training intervention (e.g., [11], [12],[13]). These effects appear to be partly mediated through enhanced expression of insulin signaling intermediates but not the activity of oxidative enzymes [14]. In addition, data from transgenic models suggest that driving a hypermuscular and more glycolytic phenotype will bestow metabolic benefits. In particular, myostatin-null agouti lethal yellow and myostatin-null obese leptin-deficient (*ob/ob*) mice exhibit markedly increased muscle mass composed of predominantly fast glycolytic fibers, decreased fat mass accumulation, and improved glycemic control compared to myostatin replete littermates [15]. Similarly, induction of a skeletal muscle specific and constitutively active Akt1 transgene in diet-induced obese mice elicited hypertrophy of fast muscle fibers and increased the expression of glycolytic enzymes [16]. These adaptations improved multiple metabolic parameters including body composition, glucose homeostasis, and energy expenditure. Thus, data from clinical studies and transgenic mice contend increasing muscle mass will improve whole-body metabolism, however, the utility of a resistance training mimetic in a model of insulin resistance has not been evaluated.

In the present study, we have compared the independent and combined effects of the PPARδ agonist and putative endurance training mimetic, GW501516, and a myostatin neutralizing antibody and potential resistance training mimetic, PF-879, on the metabolic profile of obese and insulin resistant *ob/ob* mice. We tested the hypotheses that 1) myostatin inhibition would independently improve multiple aspects of metabolism and 2) the combined effects of PPARδ activation and myostatin inhibition would confer more robust and widespread effects upon the metabolism of the *ob/ob* mouse than either agent alone.

## Materials and Methods

### Mice and Interventions

All experimental procedures were approved by the Institutional Animal Care and Use Committee of Pfizer Global Research & Development (approved protocol #15646). Male *ob/ob* mice on C57BL/6 background were purchased from the Jackson Laboratory (Bar Harbor, ME). At 5 weeks of age, 40 mice were divided into 4 groups (n = 10/group) of comparable mean body weights and fasted glucose concentrations. The groups were then randomly assigned one of the following 6-week interventions: daily oral gavage of 0.5% methylcellulose and weekly i.p. administration of saline (vehicle); daily oral gavage of the PPARδ agonist, GW501516 (10 mg/kg) (Sigma-Aldrich, St. Louis, MO); weekly i.p. administration of a myostatin neutralizing antibody, PF-879 (25 mg/kg); or GW501516 in combination with PF-879. Mice were placed in standard housing conditions and provided food (Lab Diet #5001, Lab Diet, Richmond, IN) and water *ad libitum* unless otherwise noted.

### Myostatin Monoclonal Antibody

A human monoclonal antibody, PF-879, was created in XenoMice (Abgenix, Thousand Oaks, CA) by inoculation with mature myostatin protein (R&D Systems, Minneapolis, MN) as previously described [17], [18]. In brief, hybridomas were generated using standard techniques, tested for specificity to myostatin and counter screened for growth and differentiation factor-11, transforming growth factor-β1, activin A and BMP-5 binding. For example, PF-879 inhibited myostatin-induced luciferase activity with an IC_50_ of 12 nm in a human rhabdomyosarcoma cell line (A204) transfected with the pGL3-(CAGA)_12_–luciferase reporter gene. At a 100-fold higher concentration (1.3 µm), PF-879 inhibited GDF-11-mediated activity by 30%, and was unable to inhibit either activin A- or B-induced activity.

### Body Composition and Muscle Weights

Body weight was measured weekly and lean and fat mass of individual mice were quantified at baseline and end of study using computed tomography (CT) (LaTheta, EchoMRI, Houston, TX). Abdominal subcutaneous and visceral adipose tissues were quantified by CT at the 3^rd^ lumbar vertebrae. Tissue weights were measured at the end of the study following dissection. Quadriceps muscle fiber areas were quantified as previously described [16].

### Metabolic Assessments

Non-fasted and fasted glucose concentrations were assessed 3 hours into the light cycle (9 a.m.) and following an overnight fast (16 hr), respectively. Glucose tolerance was assessed at the end of the study in fasted mice by measuring serum glucose concentrations before and time points after an i.p. bolus of glucose (0.50 g/kg) (Roche/Hitachi 912 System, Roche Diagnostics, Basel, Switzerland). Serum insulin concentrations were measured using a Meso Scale Discovery (MSD) assay (Gaithersburg, MD). Insulin sensitivity was measured following a 4 hour fast by measuring glucose concentrations before and timepoints after an i.p. bolus of insulin (4.0 mU/g). The homeostatic model of insulin resistance was calculated by multiplying the fasting insulin (ng/ml) by fasting glucose concentration and dividing by 405.

Serum free fatty acids, high density lipoproteins (HDL) and triglycerides were assessed following an overnight fast (Roche/Hitachi 912 System). Triglycerides were also measured in liver and muscle samples homogenized in 10 mM Tris (pH 7.4), 0.9% NaCl and 0.1% Triton X-100. Liver and muscle (quadriceps) glycogen concentrations were assessed as previously described [19]. Serum adiponectin was assessed using a MSD assay.

Oxygen consumption (VO_2_) and carbon dioxide production (VCO_2_) were measured for 24 hours of fed and 24 hours of fasted conditions using a comprehensive laboratory animal monitoring system (CLAMS) equipped with an Oxymax Open Circuit Calorimeter (Columbus Instruments, Columbus, OH) as previously described (n = 8/group) [17], [20]. VO_2_ and VCO_2_ were used to calculate the respiratory exchange ratio (RER) and VO_2_ and RER were used to calculate energy expenditure (kcal/hr).

### Performance Measures

Habitual physical activity of individual mice was monitored for a 48 hour period in the CLAMS using photocells as previously described [16], [17]. Mice were acclimated to a motorized treadmill (Columbus Instruments) for 3 consecutive days and physical performance was characterized by measuring running time and distance to failure using a previously described protocol modified for *ob/ob* mice (initial speed of 8 m/min, increase of 2 m/min every 3 min; 0% grade) [7], [17].

### Citrate Synthase Activity

The activity of citrate synthase in skeletal muscle lysates was measured using an assay kit (Sigma-Aldrich, CS0720) and the manufacturer's protocol.

### Metabolic Gene PCR Array

A custom RT^2^ Profiler PCR array (SABiosciences, Frederick, MD) was used to determine the expression of the metabolic genes listed in Table S1 and Table S2. Total RNA was isolated from the adipose, liver, and quadriceps muscle of 8 mice/group using TRIzol (Invitrogen, Carlsbad, CA) followed by the RNeasy Mini Kit with on-column DNase treatment (Qiagen, Valencia, CA). cDNA was prepared from total RNA (200 ng per 20 µl reaction) with the RT^2^ First Strand Kit (SABiosciences), and 50 ng of cDNA in RT^2^ qPCR Master Mix (SABiosciences) was applied to each well of the 384-well array, according to the manufacturer's instructions. After running the PCR on a 7900HT Sequence Detection System (Applied Biosystems, Foster City, CA), relative quantitation for each gene was determined by normalizing to 4 housekeeping genes (*Rps18, Actb, Gapdh, and Ppia*) and comparing to the untreated control for each tissue using the ΔΔCt method.

### Statistical Analysis

Significant differences between groups for dependent variables were tested using either single-factor (group) analysis of variance (ANOVA) or two-way (group and time) ANOVA. For single-factor and two-way ANOVAs, Tukey's multiple comparisons test was used for post hoc analyses for between-group comparisons. To assess differences between the treated and untreated groups, the ratio in the average delta CT values were compared. The bootstrap [21] was used to determine confidence intervals (via the bias-corrected method) and statistical significance [21]. Analyses were conducted using GraphPad Prism Statistical Software Version 5.0 (San Diego, CA).

## Results

### PPARδ activation attenuates body weight and fat mass accumulation and myostatin inhibition stimulates muscle growth in ob/ob mice

**Figure 1:**
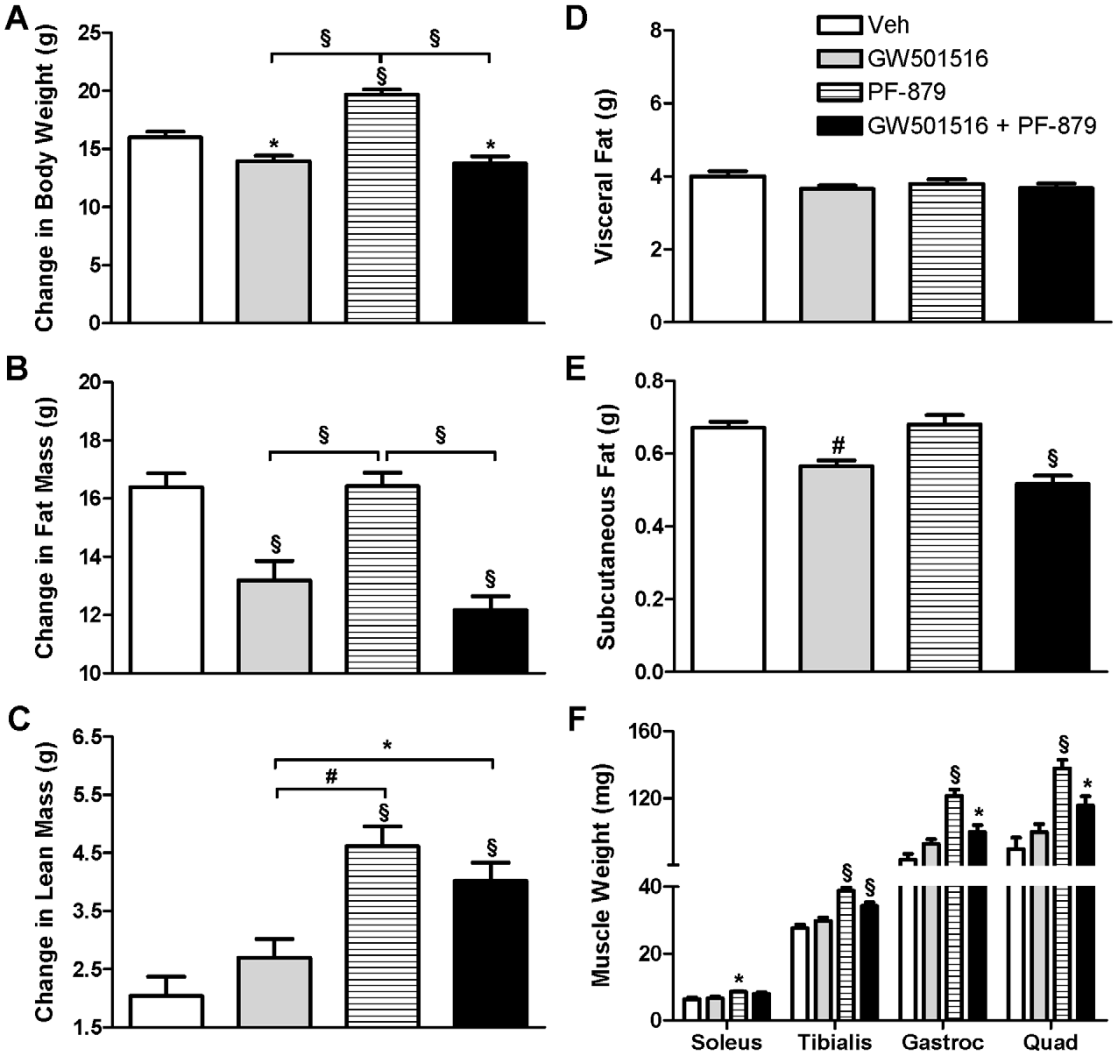
PPARδ activation and myostatin inhibition exert distinct effects on body composition. Male *ob/ob* mice were treated for 6 weeks with either vehicle (veh, open bars), PPARδ agonist GW501516 (grey bars), myostatin antibody PF-879 (hatched bars), or GW501516 in combination with PF-879 (black bars) (n = 10 mice/group). Changes in body weight (A), fat mass (B) and lean mass (C) from study start to termination are summarized. Abdominal visceral (D) and subcutaneous fat (E) were quantified for a single transverse slice acquired at the 3^rd^ lumbar vertebrae by computed tomography. Muscle weights were determined at the end of study (F). Data are represented as mean +/− SEM. *, # and § represent p<0.05, 0.01 and 0.001, respectively. See also Figure S1.

From study start to termination, daily administration of 10 mg/kg GW501516 significantly suppressed body weight and fat mass accumulation in mice compared to vehicle. In contrast, delivery of 25 mg/kg PF-879 one time per week stimulated body weight and lean mass accretion. Co-treatment of mice with GW501516 and PF-879 significantly attenuated gains in body weight and fat mass relative to vehicle, as well as augmented gains in lean mass (Figure 1A–C). There were not statistically significant differences in measured food intake among groups; however, the mice receiving GW501516 either alone or in combination with PF-879 tended to consume less (Figure S1).

**Figure 2:**
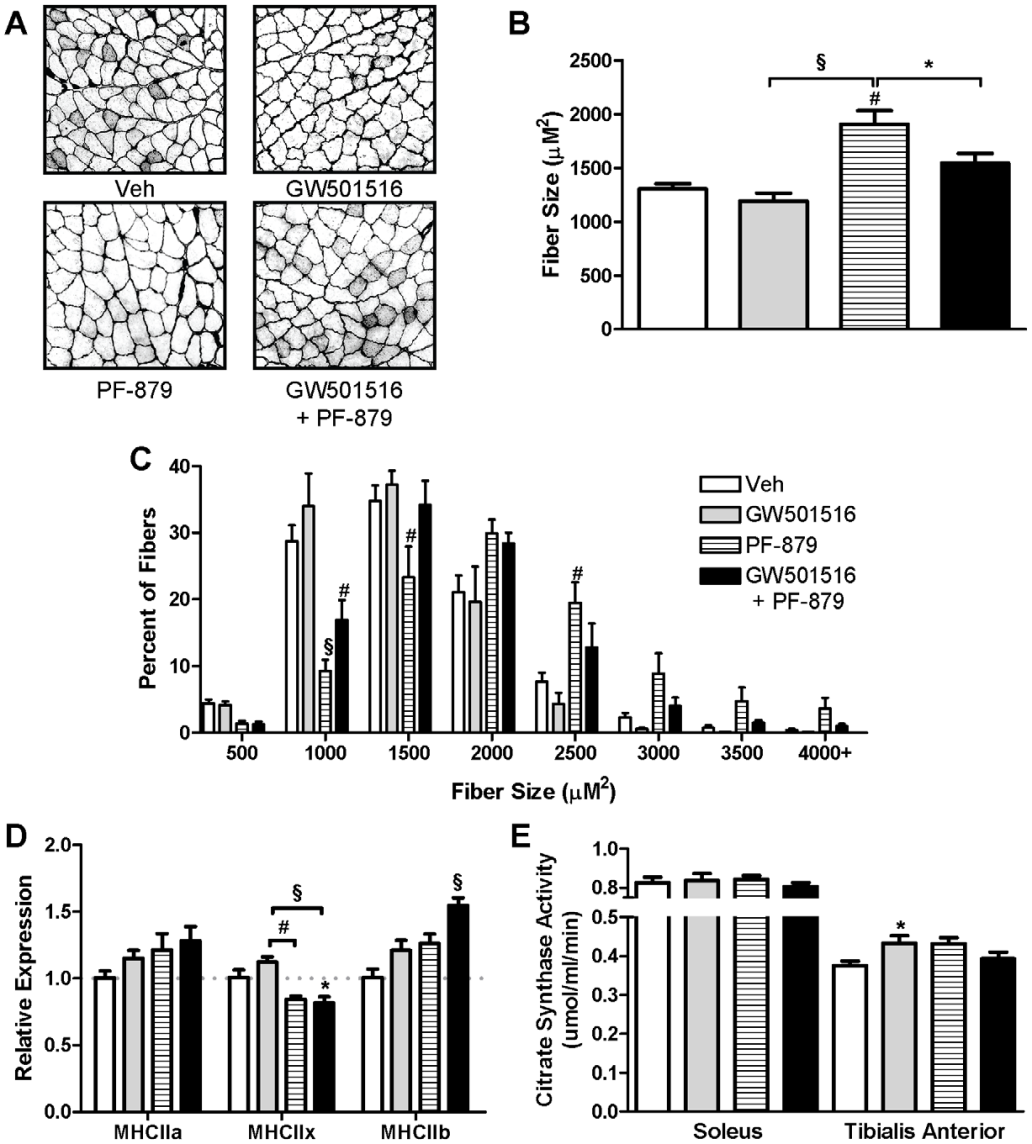
PPARδ activation increases mitochondrial activity and myostatin inhibition increases muscle fiber size in *ob/ob* mice. Male mice were treated for 6 weeks with either vehicle (veh, open bars), PPARδ agonist GW501516 (grey bars), myostatin antibody PF-879 (hatched bars), or GW501516 in combination with PF-879 (black bars). Representative cross-sectional images, the mean fiber area and size distribution of muscle fibers in the quadriceps muscle are presented in A, B and C, respectively (n = 5/group). Fold changes in quadriceps MHC mRNA expression levels relative to vehicle-treated mice are summarized (n = 5/group)(D). Citrate synthase activity was measured in soleus and tibialis anterior muscles (E). Data are represented as mean +/− SEM *, # and § indicate p<0.05, 0.01 and 0.001, respectively.

To account for differences in fat mass, individual fat pad weights and the cross-sectional area of adipocytes in the epididymal depot were measured but did not differ among groups (data not shown). Computed tomography of the abdomen, however, revealed significantly less subcutaneous fat in *ob/ob* mice treated with GW501516 either alone or in combination with PF-879 (Figure 1D and E). In regards to lean mass, administration of PF-879 and PF-879 plus GW501516 significantly increased the weight of individual muscles ≥33% and ≥20%, respectively, compared to vehicle (Figure 1F). Histological analyses of the quadriceps demonstrated that PF-879-mediated increases in mean fiber area and the percentage of larger fibers (Figure 2A–C). As shown in Figure 2D the relative abundances of MHC IIx and IIb mRNA, but not IIa, were slightly altered in the quadriceps muscle in response to the interventions, but otherwise similar across groups. Determination of citrate synthase activity revealed significantly higher levels (19%) in the tibialis anterior of GW501516-treated mice compared to vehicle (Figure 2E).

### PPARδ activation and myostatin inhibition alone and in combination improve glucose homeostasis and tolerance in ob/ob mice

**Figure 3:**
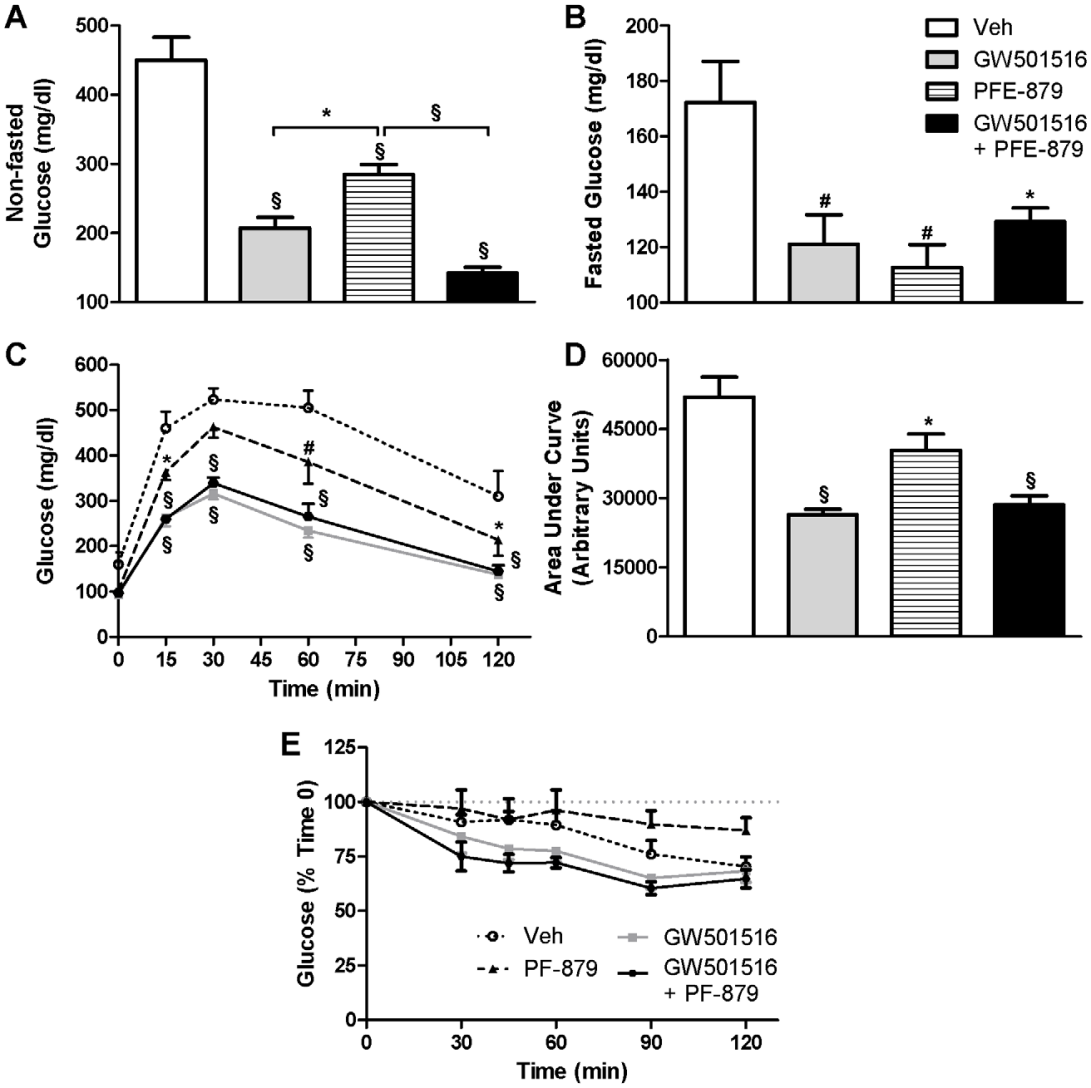
PPARδ activation and myostatin inhibition improve glucose homeostasis and tolerance in *ob/ob* mice. Following 6 weeks administration of vehicle (Veh), GW501516, PF-879, or GW501516 concurrent with PF-879, non-fasted (A) and fasted (B) glucose concentrations were measured. Blood glucose concentrations before and 2 hours after a bolus of glucose were measured following an overnight fast (C) and area under the glucose curve was calculated (D). Changes in glucose concentrations in response to a bolus of insulin were measured following a 4 hr fast (E). Data are represented as mean +/− SEM (n = 10/group). *, # and § denote p<0.05, 0.01 and 0.001, respectively.

Treatment with GW501516 and PF-879 alone significantly lowered non-fasted glucose concentrations and when administered concurrently mediated a dramatic 309 mg/dl decrease (all p<0.001) (Figure 3A). Fasted glucose concentrations were also significantly lowered by the administration of GW501516 and PF-879 alone and in combination compared to vehicle (Figure 3B). GW501516- and PF-879-treated mice exhibited lower fasting insulin concentrations compared to vehicle; however, the differences failed to reach statistical significance (mean ± SE; Veh = 19.46±2.61, GW501516 = 13.91±2.45, PF-879 = 14.76±1.43, GW501516+PF-879 = 18.58±2.72 ng/ml; ANOVA p = 0.26). The calculated homeostatic model of insulin resistance, however, was significantly improved in mice treated with PF-879 (Veh = 8.75±1.62; GW501516 = 4.50±0.79; PF-879 = 4.44±0.59; GW501516+PF-879 = 6.40±0.93; p<0.05).

All 3 interventions significantly improved glucose tolerance in the *ob/ob* mice; however, GW501516 administration alone and concurrent with PF-879 more dramatically suppressed glucose excursion from baseline and facilitated disposal following the glucose bolus (Figure 3C and 3D). As illustrated in Figure 3E, no statistical differences in glucose lowering in response to an insulin bolus were noted between the groups of *ob/ob* mice. Finally, GW501516 and GW501516 plus PF-879 significantly decreased liver glycogen concentrations, while PF-879 modestly but insignificantly lowered glycogen content in skeletal muscle (Figure S2).

### Myostatin Inhibition increases oxygen consumption and basal metabolic rate in ob/ob mice

**Figure 4:**
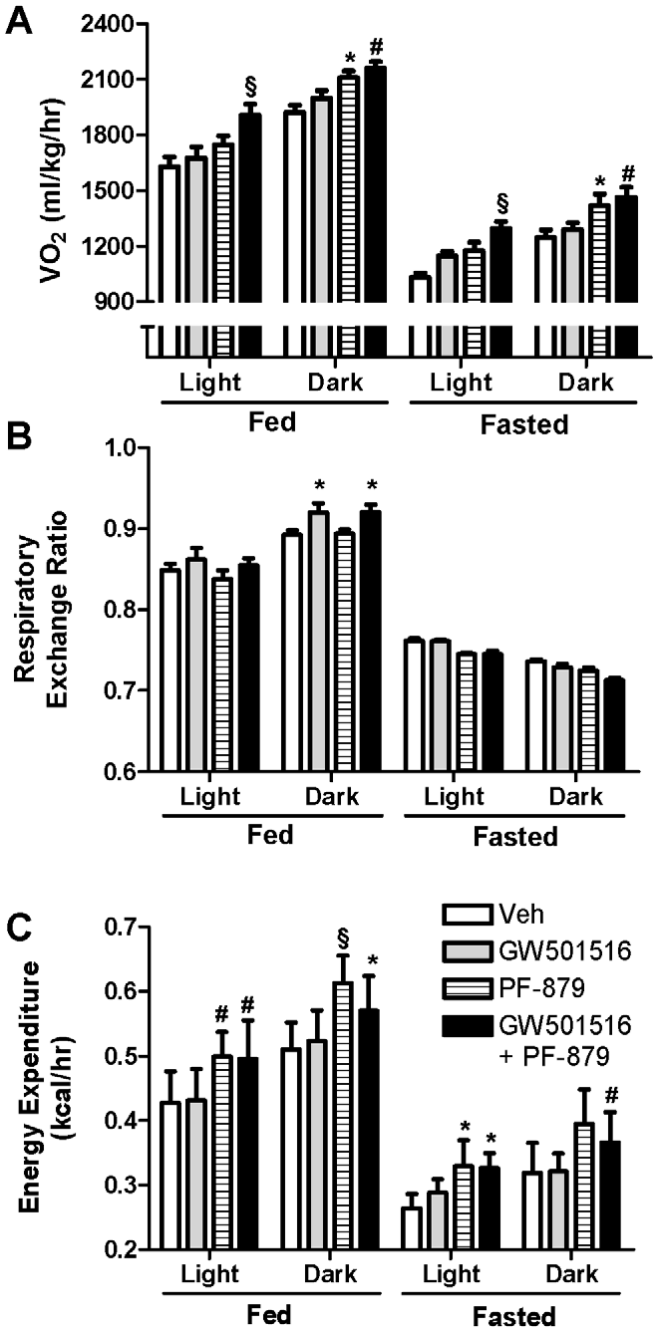
Myostatin inhibition increases oxygen consumption and energy expenditure in *ob/ob* mice. Indirect calorimetry was performed following 6 weeks administration of vehicle (veh), GW501516, PF-879, or GW501516 in combination with PF-879 to quantify oxygen consumption (VO_2_) (A) and carbon dioxide production to calculate the respiratory exchange ratio (B) and energy expenditure (C) in *ob/ob* mice. Data are represented as mean +/− SEM (n = 8/group). *, # and § represent p<0.05, 0.01 and 0.001, respectively.

Oxygen consumption (VO_2_) and energy expenditure were significantly increased during fed and fasted states in mice treated with PF-879 and PF-879 plus GW501516 compared to vehicle. No effect of GW501516 treatment alone was noted (Figure 4A and C). Meanwhile, the respiratory exchange ratio (RER) was modestly elevated during the fed state in mice treated with GW501516 alone and concurrent with PF-879 (both p<0.05) (Figure 4B).

### PPARδ activation and myostatin inhibition affect muscle triglycerides, lipid profiles and adiponectin levels in ob/ob mice

**Figure 5:**
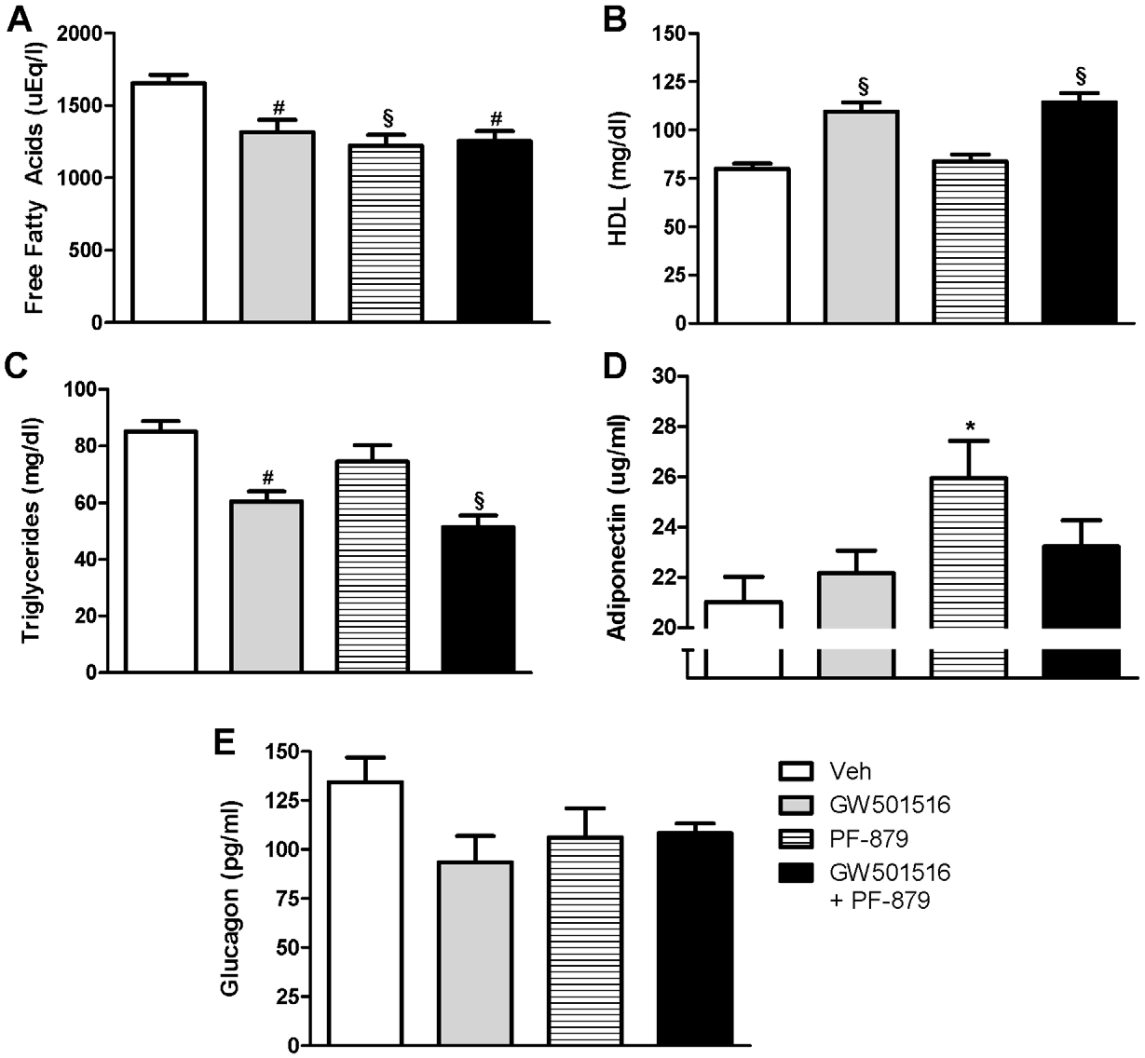
PPARδ activation and myostatin inhibition improve the lipid profile and alter adiponectin in *ob/ob* mice. Serum lipids were analyzed following 6 weeks treatment with vehicle (veh), GW501516, PF-879, or GW501516 in combination with PF-879. Free fatty acids (A), high density lipoproteins (HDL) (B) and triglycerides (C) are illustrated. Circulating adiponectin levels (D) and glucagon concentrations (E) are also summarized. Data are represented as mean +/− SEM (n = 10/group). *, # and § indicate p<0.05, 0.01 and 0.001, respectively. See also Figure S2.

Compared to vehicle, 6-weeks of GW501516 and PF-879 administration, either alone or concurrently, significantly decreased muscle triglycerides and serum free fatty acids (FFAs) in *ob/ob* mice, but had no effect on liver triglycerides (Figure 5A and Figure S2). Moreover, GW501516 treatment significantly decreased triglyceride and increased HDL serum concentrations. The effects of PF-879-treatment on triglyceride and HDL levels were not statistically significant, but its co-administration did not attenuate GW501516-mediated effects (Figure 5B and C). PF-879 independently increased serum adiponectin levels by 24% in the *ob/ob* mice compared to vehicle. No treatment effects were observed on plasma glucagon concentrations (Figure 5E).

### Myostatin inhibition increases physical activity and performance in ob/ob mice

**Figure 6:**
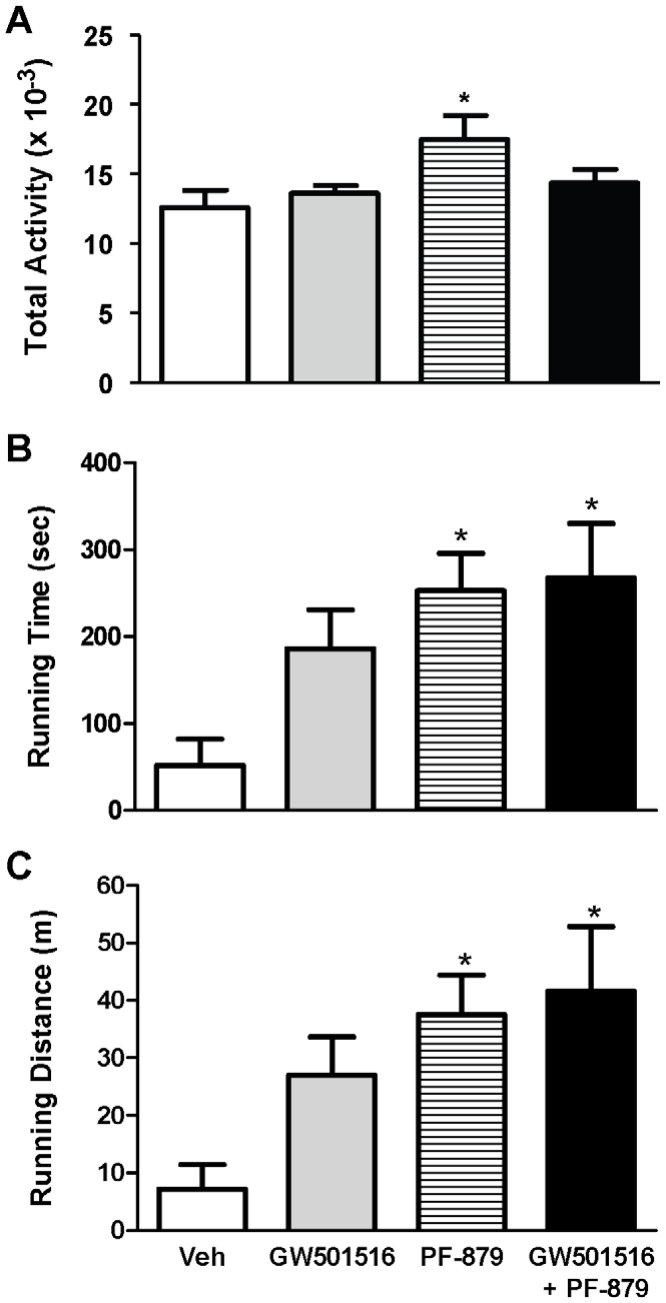
Myostatin inhibition increases habitual physical activity and improves performance in *ob/ob* mice. After 6 weeks of treatment with vehicle (veh), GW501516, PF-879, or GW501516 in combination with PF-879, habitual physical activity was monitored and summed for a 48 hour period (A) (n = 8/group). At study end, the time (B) and distance (C) mice were able to comply with a performance protocol on a motorized treadmill was also measured (n = 10/group). Data are represented as mean +/− SEM. * represents p<0.05.

To assess habitual physical activity in the *ob/ob* mice, horizontal movement was monitored over a 48 hour period and was increased by nearly 40% in mice treated with PF-879 compared to vehicle (p<0.05) (Figure 6A). No effect of GW501516 was observed. Moreover, running time and distance to failure in mice administered PF-879 alone as well as in combination with GW501516 were both increased >3.8- and >4.1-fold, respectively, compared to vehicle (all p<0.05) (Figure 6B and C). Mice receiving GW501516 also exhibited increased running time (2.5-fold) and distance (2.7-fold) to failure relative to those receiving vehicle; however, these differences were not statistically significant.

### PPARδ activation and myostatin inhibition differentially affect metabolic gene expression in ob/ob mice

**Figure 7:**
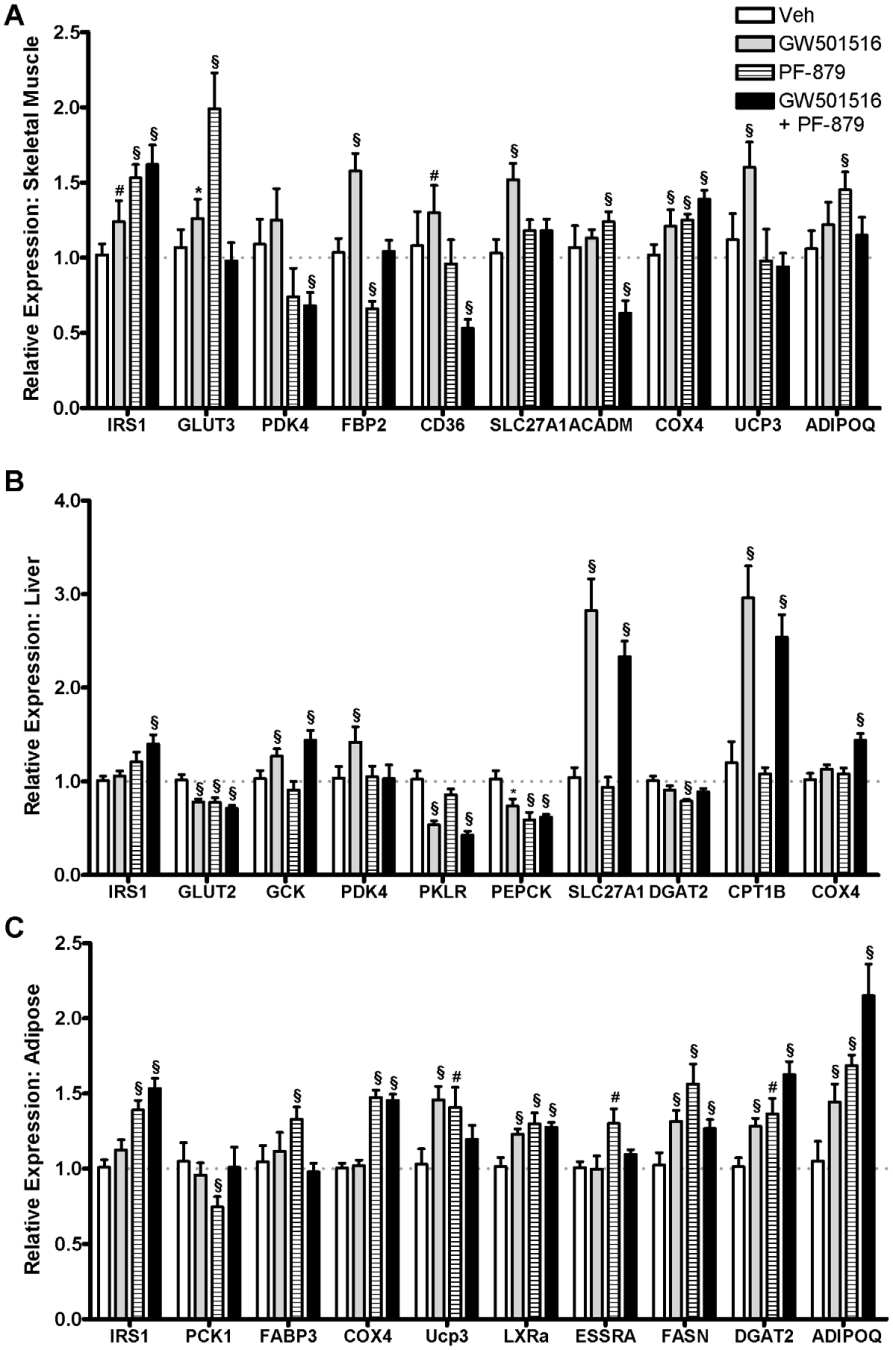
The effects of PPARδ activation and myostatin inhibition on metabolic gene expression in *ob/ob* mice. Following 6 weeks of treatment with either vehicle (Veh), GW501516, PF-879 or GW501516 plus PF-879, mRNA was isolated from skeletal muscle, liver and adipose tissue (A, B and C, respectively) of *ob/ob* mice and quantified by a low-density array card. Fold changes compared to vehicle were calculated. Data are represented as mean +/− SEM (n = 8/group). *, # and § represent differences in expression of >20% relative to control and p<0.05, 0.01 and 0.001, respectively. See also Table S1.

The effects of 6-weeks treatment with GW501516 and PF-879 alone and in combination on metabolic gene expression in muscle, liver and adipose tissue are detailed in Table S1 and subsets are illustrated in Figure 7. In skeletal muscle, GW501516 significantly increased expression of genes regulating fatty acid transport at the cell surface (CD36, SLC27A1) and mitochondrial membrane (CPT1B), mitochondrial uncoupling (UCP2 and UCP3), and oxidative metabolism (COX4I1) compared to vehicle. More consistent and robust effects of GW501516 on these and related genes were measured in the liver, while increased expression of adiponectin and genes involved in fatty acid synthesis (DGAT2, FASN) were noted in adipose. In skeletal muscle, PF-879 increased the expression of genes involved in insulin signaling (IRS1) and glucose transport (GLUT3) and decreased the expression of PEPCK and FBP2 compared to vehicle as to favor glucose utilization. Increased expression of adiponectin was also detected. In liver, few changes were noted in response to PF-879, while changes in adipose were largely similar to those observed in skeletal muscle. In contrast to the physiological effects of GW501516 and PF-879 being highly conserved with co-administration, the changes in metabolic gene expression in skeletal muscle were entirely distinct. In particular, the expression of several factors in insulin signaling and glucose transport and metabolism (i.e., IRS2, GLUT4, PDK4 and PEPCK) and fatty acid transport (CD36 and FABP3) and oxidation (ACADM, PGC1β) were significantly decreased in the combination group. In the liver, the effects of GW501516 were highly conserved when delivered concurrently with PF-879, and of note, FGF21 was significantly increased. Changes observed in the expression of genes of fatty acid and oxidative metabolism in adipose of mice treated with GW501516 and PF-879 independently were largely reflected in the combination group.

## Discussion

In the present study, we show for the first time that postnatal inhibition of myostatin improves the metabolic profile of obese insulin resistant *ob/ob* mice. Administration of a neutralizing antibody to myostatin, PF-879, led to hypertrophy of skeletal muscle in a manner similar to resistance training. PF-879 improved metrics of glucose homeostasis, energy expenditure and physical function in the absence of beneficial changes in body weight, fat mass or liver triglycerides. In several instances the effects of PF-879 were distinct from, and in others complimentary to, those induced by the PPARδ agonist and reputed endurance training mimetic, GW501516.

The discoveries of myostatin and the double-muscled phenotype of myostatin-null mice [22] have generated great interest in devising biotherapeutic approaches (e.g., myostatin peptides, propeptides and neutralizing antibodies) to disrupt myostatin signaling. These agents have unequivocally increased muscle mass in murine models of health and neuromuscular disease and validated myostatin as a key regulator of muscle maintenance in adulthood (e.g., [23], [24]) and even later life [17]. In the present study, PF-879 markedly increased lean mass in morbidly obese mice by driving the growth of both fast-twitch glycolytic and slow-twitch oxidative fiber-enriched skeletal muscles (e.g., tibialis anterior and soleus, respectively). In contrast to transgenic knockout approaches to myostatin [15], [25] and treatment of obese mice with GW501516 (here and previous reports [8], [26]), PF-879-administration did not attenuate fat mass or body weight gain. This finding is consistent with a previous report of a different neutralizing antibody to myostatin [23], [27]. Even so, the PF-879-mediated increases in skeletal muscle mass translated into physiological and functional improvements as reflected by increases in VO_2_ and energy expenditure, habitual physical activity and exercise capacity. These effects were unique to PF-879 and with the exception of habitual physical activity, largely conserved when co-administered with GW501516. Moreover, the salutary effects of GW501516 on body weight and fat mass were equally robust in mice co-administered PF-879. These data indicate that postnatal inhibition of myostatin increases muscle mass in a manner similar to resistance training and unique to GW501516, and leads to physiological and functional improvements.

Despite the very distinct effects of PPARδ activation and myostatin inhibition on body weight and composition, both GW501516 and PF-879 improved multiple metabolic parameters in the obese, hyperinsulinemic and insulin resistant mice. Administration of GW501516 and PF-879 alone and concurrently significantly decreased fasted (all ≤126 mg/dl) and non-fasted glucose concentrations, and the two interventions alone tended to lower fasting insulin concentrations. The impact of PF-879 on both glycemia and insulinemia was further highlighted in an improved HOMA-IR. Meaningful changes in the response to exogenously administered glucose were observed in all three treatment arms and most notably in the two receiving GW501516. Consistent with promoting oxidation of lipids in PPARδ-expressing tissues, GW501516 decreased circulating triglycerides and FFAs and muscle triglycerides. PF-879 also decreased serum free fatty acids and muscle triglycerides, and notably, increased serum concentrations of adiponectin. The increase in muscle glycogen and decrease in liver triglycerides experienced by the combination group suggest the salutary effects of GW501516 and PF-879 on glucose transport and fatty acid oxidation are synergistic and may further enhance systemic metabolism. Overall, the data 1) for the first time show that postnatal inhibition of myostatin can improve metabolic homeostasis in obese insulin resistant mice; 2) confirm the previously reported endurance training-like effects of PPARδ activation on metabolic parameters [8], [26]; and 3) advance the notion that strategies to increase skeletal muscle mass in a resistance training manner improve multiple aspects of whole body metabolism.

The mechanism by which postnatal myostatin inhibition improves metabolic homeostasis is particularly intriguing. Similar to resistance training, conditional expression of a constitutively active Akt1-transgene [16], transgenic deletion of myostatin [15], [25], and expression of a dominant negative ActRIIb receptor in skeletal muscle [25], PF-879 robustly increased skeletal muscle mass and improved glucose homeostasis and tolerance by increasing the responsiveness to similar if not lower concentrations of insulin; however, there are also several key distinctions. First, the metabolic improvements observed in response to PF-879 occurred independent of decreases in body weight, adiposity or hepatic lipid accumulation. In contrast, deletion or loss of function mutations in myostatin, and conditional muscle-specific overexpression of Akt1, clearly protect against or reverse diet-induced obesity and hepatic lipid deposition [16], [25], [28]. Moreover, a soluble ActRIIB/Fc fusion protein administered to high fat-fed mice also increased muscle mass at 4 weeks, but did not affect either fat mass or glucose concentrations until 10 weeks [29]. A study of longer duration or conducted in a less severe model of metabolic dysfunction may reveal similar effects by PF-879, however, they do not account for the positive metabolic changes observed here. Second, we observed increased energy expenditure in mice treated with PF-879 (similar to *Akt1* transgenic mice [16]) and no difference in RER compared to vehicle-treated mice. In contrast, myostatin null mice exhibit reduced oxygen consumption relative to body weight and increased carbohydrate utilization as determined by a higher RER (consequent to the higher number and percentage of fast-twitch glycolytic fibers [22], [30]) [15], [25]. We posit that the systemic increase in skeletal muscle mass and resultant increase in energy demand and expenditure largely account for PF-879-mediated improvements in whole-body metabolism. Third, compared to vehicle- and GW501516-treated mice, mice receiving PF-879 had significantly increased serum adiponectin concentrations. This is contrast to the reduced serum concentrations of adiponectin in mice with either a loss of function mutation in myostatin or mice overexpressing the inhibitory myostatin propeptide on a standard diet [28], [31], but consistent with mice receiving a soluble ActRIIb/Fc fusion protein that also exhibited a reduction in hepatic glucose production [29]. We conclude the increase in adiponectin in part contributes to the metabolic improvements observed in mice administered PF-879. Collectively, these data indicate that PF-879-mediated metabolic improvements in this model are largely consequent to the increase in skeletal muscle- the primary site of insulin-mediated glucose disposal that also accounts for a substantial percentage of energy expenditure- and possibly increased adiponectin, but cannot be explained by a reduction in fat mass or liver triglycerides.

To further explore the impact of GW501516 and PF-879 on the metabolic profile of muscle, liver and adipose, we examined and compared the expression of key mediators of glucose and fatty acid metabolism. As indicated by the results and in agreement with previous reports [26], [32], GW501516 increased the expression of genes of oxidative metabolism in skeletal muscle. These changes were manifest in increased citrate synthase activity and lowered muscle triglycerides and likely account for the improvements in insulin responsiveness and glucose homeostasis. Intriguingly, despite profound changes in skeletal muscle mass, the gene expression profile was largely unaffected in response to PF-879. This is in contrast to the overtly glycolytic skeletal muscle phenotype and genotype induced by deletion of myostatin or conditional overexpression of *Akt1* [16],[25], though the modest changes in IRS1, GLUT3 and ADIPOQ in response to PF-879 may contribute to improved insulin sensitivity [33], and decreases in PEPCK and FBP2 may promote glucose utilization and account for the modest decrease in muscle glycogen content [34], [35]. The broad agreement between the 3 interventions with respect to changes in gene expression in adipose implies that these effects may simply be secondary to the improved metabolic profile in the *ob/ob* mice. Overall, these data suggest that postnatal inhibition of myostatin, unlike deletion of myostatin, overexpression of Akt1 or administration of a PPARδ agonist, does not remarkably alter the expression of key metabolic genes in muscle, liver or adipose tissue as a means to improve systemic metabolism.

The potential benefit of myostatin blockade for T2DM has been substantiated by several recent studies. First, increased myostatin expression and more impressively secretion has been observed in muscle and adipose tissue samples derived from obese and extremely obese women, and increased circulating levels of myostatin in this cohort were found to be correlated with insulin resistance [36]. Second, myostatin expression in skeletal muscle was significantly decreased in response to weight loss in obese patients undergoing gastric bypass surgery and associated with increased insulin action [37], [38]. Third, increased expression of *myostatin* was recently detected in skeletal muscle biopsies of healthy but at risk first degree relatives of patients with T2DM in concert with genes of the insulin signaling pathway [39]. Coupled with the findings of the present study, these data suggest that myostatin likely contributes to alterations in skeletal muscle quantity, quality and metabolism in conditions of obesity and T2DM and a pharmacological strategy to disrupt myostatin signaling may potentially prevent, attenuate or reverse their progression.

In summary, the data provide convincing evidence that a neutralizing antibody to myostatin not only promotes gains in muscle mass similar to resistance training, but improves fasting and fed glucose concentrations, glucose tolerance and lipid profiles in obese insulin resistant *ob/ob* mice. In addition, PF-879 increased energy expenditure and measures of physical function and did not diminish the unique effects of GW501516 on body weight, adiposity or serum triglycerides. These data further suggest clinical and pharmacological strategies to increase muscle mass, and not necessarily oxidative capacity, may effectively counter insulin resistance and T2DM.

## Supporting Information

Figure S1 Effects of PPARδ activation and myostatin inhibition on food intake and body weight in *ob/ob* mice. Food intake (A) and body weight (B) were monitored weekly for individual mice for 6 weeks while treated with either vehicle (Veh), GW501516, PF-879 or GW501516 plus PF-879. n = 10/group and *, # and § represent p<0.05, 0.01 and 0.001, respectively.
(0.53 MB TIF)

Figure S2 Effects of PPARδ activation and myostatin inhibition on skeletal muscle and liver glycogen and triglyceride content in *ob/ob* mice. Male *ob/ob* mice were treated for 6 weeks with vehicle (Veh), GW501516, PF-879 or GW501516 plus PF-879. At study termination, the concentrations of glycogen and triglycerides were measured in skeletal muscle (A and B) and liver (C and D) (n = 8-10/group). *, # and § represent p<0.05, 0.01 and 0.001, respectively.
(0.58 MB TIF)

Table S1 Fold changes (mean (standard error))^A^ in metabolic gene expression in the muscle, liver and adipose of *ob/ob* mice treated for 6 weeks with either a PPARδ agonist (GW501516), a neutralizing antibody to myostatin (PF-879) or both (GW+PF) relative to a vehicle control.
(0.13 MB DOC)

Table S2 Symbols, aliases, and official names of genes examined by PCR array.
(0.08 MB DOC)
