# Supplementary figures and images for: Postnatal PPARδ Activation and Myostatin Inhibition Exert Distinct yet Complimentary Effects on the Metabolic Profile of Obese Insulin-Resistant Mice
Source: PLoS One. 2010 Jun 25;5(6):e11307. doi: 10.1371/journal.pone.0011307 (PMC2892469; doi:10.1371/journal.pone.0011307)

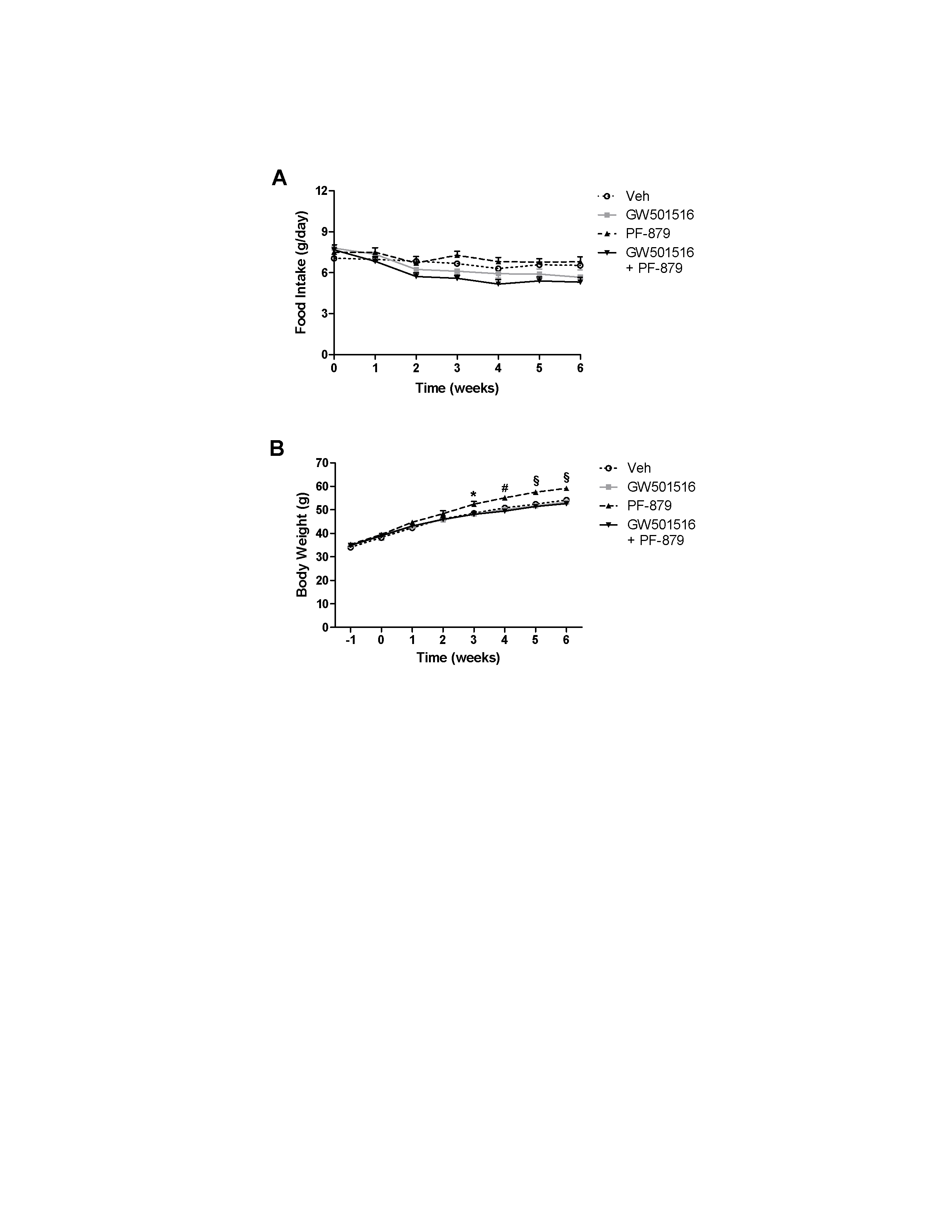

Supplement: Figure S1 — Effects of PPARδ activation and myostatin inhibition on food intake and body weight in ob/ob mice. Food intake (A) and body weight (B) were monitored weekly for individual mice for 6 weeks while treated with either vehicle (Veh), GW501516, PF-879 or GW501516 plus PF-879. n = 10/group and *, # and § represent p<0.05, 0.01 and 0.001, respectively. (0.53 MB TIF) [file pone.0011307.s001.tif]

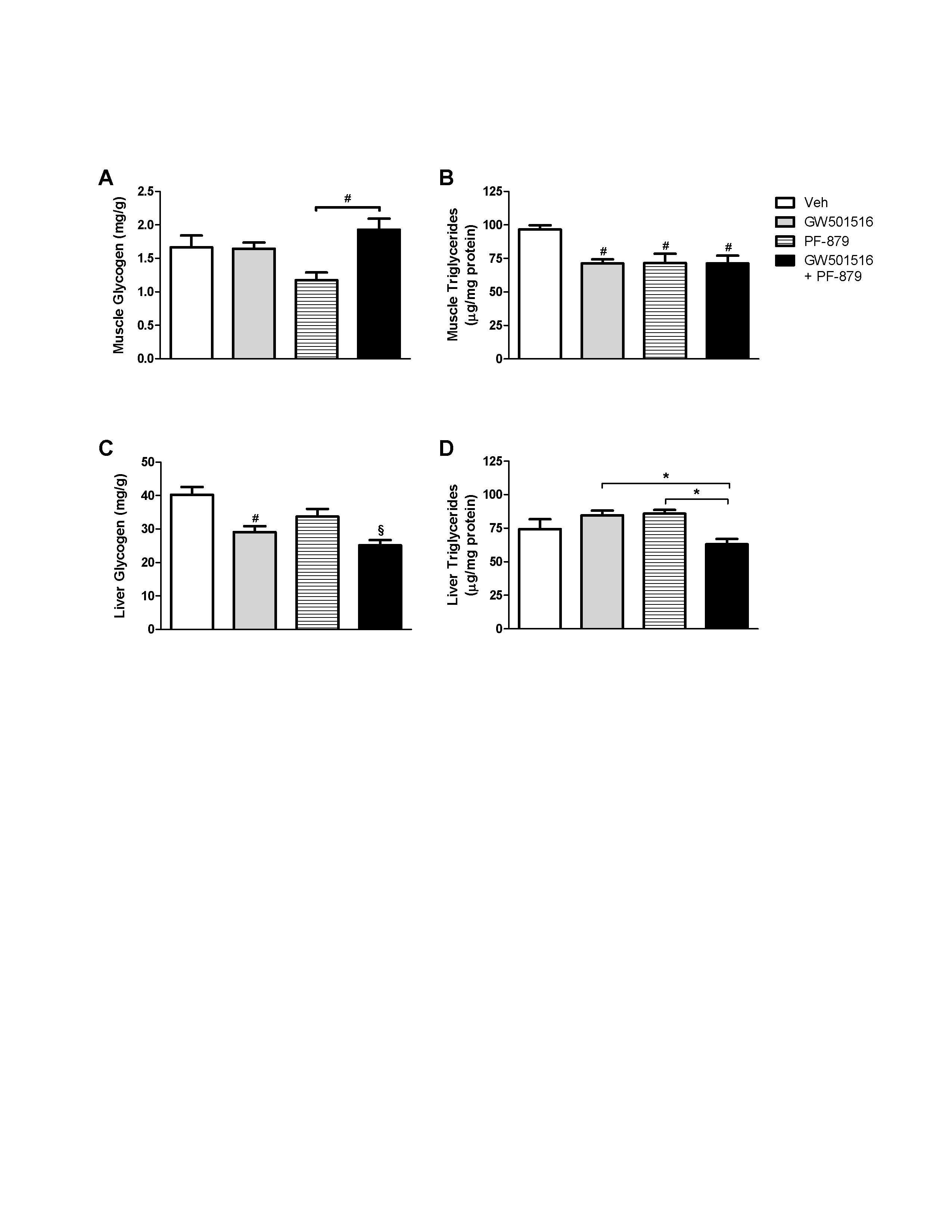

Supplement: Figure S2 — Effects of PPARδ activation and myostatin inhibition on skeletal muscle and liver glycogen and triglyceride content in ob/ob mice. Male ob/ob mice were treated for 6 weeks with vehicle (Veh), GW501516, PF-879 or GW501516 plus PF-879. At study termination, the concentrations of glycogen and triglycerides were measured in skeletal muscle (A and B) and liver (C and D) (n = 8-10/group). *, # and § represent p<0.05, 0.01 and 0.001, respectively. (0.58 MB TIF) [file pone.0011307.s002.tif]
