# Supplementary material for: Postnatal PPARδ Activation and Myostatin Inhibition Exert Distinct yet Complimentary Effects on the Metabolic Profile of Obese Insulin-Resistant Mice
Source: PLoS One. 2010 Jun 25;5(6):e11307. doi: 10.1371/journal.pone.0011307 (PMC2892469; doi:10.1371/journal.pone.0011307)
Supplement: Table S1 — Fold changes (mean (standard error))A in metabolic gene expression in the muscle, liver and adipose of ob/ob mice treated for 6 weeks with either a PPARδ agonist (GW501516), a neutralizing antibody to myostatin (PF-879) or both (GW+PF) relative to a vehicle control. (0.13 MB DOC) [file pone.0011307.s003.doc]

|  | **Muscle** | | | **Liver** | | | **Adipose** | | |
| --- | --- | --- | --- | --- | --- | --- | --- | --- | --- |
| **Genes^B^** | **GW501516** | **PF-879** | **GW + PF** | **GW501516** | **PF-879** | **GW + PF** | **GW501516** | **PF-879** | **GW + PF** |
| **Insulin Signaling and Glucose Metabolism** | | | | | | | | | |
| Irs1 | 1.24 (0.14)^#^ | 1.53 (0.09)**^§^** | 1.62 (0.13)**^§^** | 1.06 (0.05) | 1.21 (0.11) | 1.40 (0.10)**^§^** | 1.12 (0.07) | 1.39 (0.07)**^§^** | 1.53 (0.07)**^§^** |
| Irs2 | 0.90 (0.09) | 1.14 (0.08) | 0.74 (0.05)**^§^** | 1.50 (0.33)***** | 1.22 (0.23) | 1.43 (0.17)**^§^** | 0.92 (0.04) | 1.26 (0.11)***** | 0.86 (0.06) |
| Glut1 | 0.98 (0.09) | 1.08 (0.06) | 0.86 (0.05) | 0.97 (0.06) | 0.96 (0.07) | 0.95 (0.06) | 0.87 (0.05) | 0.99 (0.04) | 0.85 (0.05) |
| Glut2 | - | - | - | 0.78 (0.03)**^§^** | 0.78 (0.05)**^§^** | 0.71 (0.03)**^§^** | 0.81 (0.06) | 0.94 (0.18) | 0.85 (0.09) |
| Glut3 | 1.26 (0.13)* | 1.99 (0.24)**^§^** | 0.98 (0.12) | - | - | - | 0.92 (0.10) | 0.96 (0.07) | 0.83 (0.04) |
| Glut4 | 0.88 (0.07) | 0.91 (0.04) | 0.80 (0.04)**^§^** | 0.89 (0.08) | 1.05 (0.19) | 0.89 (0.08) | 0.81 (0.04) | 1.00 (0.04) | 1.04 (0.08) |
| GK | 0.89 (0.10) | 0.79 (0.08)**^#^** | 0.72 (0.07)**^§^** | 1.27 (0.08)**^§^** | 0.91 (0.09) | 1.44 (0.11)**^§^** | - | - | - |
| Hk2 | 1.00 (0.08) | 0.89 (0.05) | 0.84 (0.05) | 1.34 (0.14)**^#^** | 1.04 (0.11) | 1.04 (0.13) | 0.92 (0.04) | 0.92 (0.07) | 0.94 (0.08) |
| Pfkm | 1.24 (0.12)* | 1.23 (0.13)* | 0.90 (0.13) | 0.81 (0.03) | 0.98 (0.09) | 0.94 (0.07) | 1.16 (0.03) | 1.27 (0.09)**^#^** | 1.04 (0.07) |
| Pfkl | - | - | - | 1.08 (0.10) | 1.00 (0.09) | 1.14 (0.13) | 1.04 (0.07) | 1.13 (0.06) | 1.01 (0.06) |
| Pdk4 | 1.25 (0.21) | 0.74 (0.19) | 0.68 (0.09)**^§^** | 1.42 (0.16)**^§^** | 1.05 (0.12) | 1.03 (0.15) | 1.24 (0.15)* | 1.02 (0.11) | 0.88 (0.07) |
| Pepck | 1.18 (0.32) | 0.68 (0.07)**^§^** | 0.52 (0.05)**^§^** | 0.74 (0.08)* | 0.59 (0.08)**^§^** | 0.62 (0.03)**^§^** | 0.96 (0.08) | 0.74 (0.07)**^§^** | 1.01 (0.13) |
| G6Pc | - | - | - | 1.07 (0.18) | 1.02 (0.18) | 1.31 (0.29) | - | - | - |
| Fbp2 | 1.58 (0.12)**^§^** | 0.66 (0.05)**^§^** | 1.04 (0.07) | - | - | - | 1.18 (0.09) | 0.90 (0.10) | 1.15 (0.16) |
| **Fatty Acid Metabolism** | | | | | | | | | |
| Cd36 | 1.30 (0.18)* | 0.96 (0.16) | 0.53 (0.06)**^§^** | 1.19 (0.15) | 1.03 (0.12) | 1.03 (0.17) | 1.12 (0.09) | 1.08 (0.10) | 0.59 (0.04)**^§^** |
| Slc27a1 | 1.52 (0.11)**^§^** | 1.18 (0.07) | 1.18 (0.08) | 2.82 (0.34)**^§^** | 0.94 (0.11) | 2.33 (0.17)**^§^** | 1.06 (0.04) | 1.02 (0.06) | 0.89 (0.05) |
| Fabp3 | 0.90 (0.07) | 1.04 (0.11) | 0.77 (0.06)**^#^** | - | - | - | 1.12 (0.13) | 1.33 (0.08)**^§^** | 0.98 (0.06) |
| Acc1 | 1.30 (0.10)* | 1.28 (0.09)**^§^** | 0.87 (0.06) | 1.24 (0.15) | 1.26 (0.08)**^§^** | 0.99 (0.13) | 1.20 (0.11)* | 1.49 (0.11)**^§^** | 1.35 (0.05)**^§^** |
| Acc2 | 1.13 (0.08) | 1.07 (0.05) | 0.86 (0.06) | 1.73 (0.15)**^§^** | 1.18 (0.09)* | 1.46 (0.13)**^§^** | 0.97 (0.04) | 0.96 (0.04) | 0.83 (0.05) |
| Acadm | 1.13 (0.06) | 1.24 (0.07)**^§^** | 0.63 (0.09)**^§^** | 1.19 (0.14) | 0.81 (0.12) | 1.08 (0.10) | 1.01 (0.04) | 1.16 (0.05) | 0.77 (0.16) |
| Cpt1a | - | - | - | 1.34 (0.13)**^#^** | 0.80 (0.08)* | 1.16 (0.17) | 1.30 (0.1)**^§^** | 1.29 (0.22) | 0.70 (0.09)**^§^** |
| Cpt1b | 1.28 (0.07)**^§^** | 1.10 (0.06) | 0.97 (0.06) | 2.96 (0.34)**^§^** | 1.08 (0.07) | 2.54 (0.24)**^§^** | 1.04 (0.05) | 1.14 (0.06) | 0.95 (0.06) |
| Gpat1 | 1.11 (0.06) | 0.96 (0.09) | 0.76 (0.06)**^#^** | 1.37 (0.16)**^#^** | 1.18 (0.1) | 1.12 (0.12) | 1.13 (0.05) | 1.01 (0.04) | 0.87 (0.05) |
| Dgat1 | 0.96 (0.06) | 1.08 (0.04) | 0.85 (0.04) | 0.91 (0.05) | 0.79 (0.02)**^§^** | 0.89 (0.04) | 0.97 (0.04) | 1.02 (0.05) | 0.97 (0.04) |
| Dgat2 | 1.08 (0.11) | 1.14 (0.06) | 0.87 (0.06) | 0.98 (0.05) | 1.06 (0.13) | 1.07 (0.11) | 1.28 (0.05)**^§^** | 1.37 (0.10)**^#^** | 1.63 (0.09)**^§^** |
| Fasn | - | - | - | 1.14 (0.13) | 1.09 (0.11) | 1.45 (0.22)* | 1.31 (0.07)**^§^** | 1.57 (0.13)**^§^** | 1.27 (0.06)**^§^** |
| Chrebp | 1.00 (0.03) | 0.94 (0.10) | 0.69 (0.02)**^§^** | 0.84 (0.07) | 0.89 (0.13) | 0.86 (0.10) | 1.12 (0.05) | 1.03 (0.09) | 0.86 (0.06) |
| Srebp1 | 1.02 (0.04) | 1.25 (0.05)**^§^** | 0.84 (0.05) | 1.18 (0.11) | 0.93 (0.11) | 1.21 (0.07)**^§^** | 1.06 (0.02) | 1.11 (0.04) | 1.07 (0.05) |
| Lxrα | 1.00 (0.10) | 1.11 (0.04) | 0.90 (0.05) | 0.94 (0.05) | 0.93 (0.05) | 1.12 (0.11) | 1.23 (0.04)**^§^** | 1.30 (0.07)**^§^** | 1.28 (0.03)**^§^** |
| Pklr | - | - | - | 0.54 (0.04)**^§^** | 0.86 (0.06) | 0.43 (0.04)**^§^** | 0.27 (0.05)**^§^** | 0.91 (0.44) | 0.68 (0.28) |

|  | **Muscle** | | | **Liver** | | | **Adipose** | | |
| --- | --- | --- | --- | --- | --- | --- | --- | --- | --- |
|  | **GW501516** | **PF-879** | **GW + PF** | **GW501516** | **PF-879** | **GW+PF** | **GW501516** | **PF-879** | **GW+PF** |
| **Mitochondrial Biogenesis & Oxidative Metabolism** | | | | | | | | | |
| Pgc1α | 1.06 (0.07) | 0.93 (0.07) | 0.88 (0.16) | 0.90 (0.07) | 0.78 (0.10) | 0.98 (0.10) | - | - | - |
| Pgc1β | 0.94 (0.05) | 0.86 (0.05) | 0.76 (0.05)**^§^** | - | - | - | - | - | - |
| Pparα | 1.09 (0.10) | 0.99 (0.12) | 0.94 (0.10) | 1.17 (0.09)* | 0.86 (0.10) | 1.05 (0.07) | 0.75 (0.04)**^§^** | 0.82 (0.09) | 0.87 (0.11) |
| Pparδ | 0.99 (0.05) | 1.05 (0.03) | 0.89 (0.07) | 0.88 (0.05)* | 0.86 (0.05) | 1.00 (0.05) | 1.03 (0.03) | 1.29 (0.07)**^§^** | 1.12 (0.05) |
| Esrra | 1.17 (0.09) | 1.20 (0.12)* | 0.97 (0.05) | 0.98 (0.08) | 0.86 (0.09) | 0.92 (0.08) | 1.00 (0.09) | 1.30 (0.10)**^#^** | 1.09 (0.03) |
| Mfn2 | 0.99 (0.08) | 1.02 (0.07) | 0.81 (0.02) | 1.93 (0.13)**^§^** | 1.76 (0.11)**^§^** | 1.90 (0.17)**^§^** | 0.94 (0.04) | 1.05 (0.06) | 0.91 (0.04) |
| Ucp1 | - | - | - | - | - | - | - | - | - |
| Ucp2 | 1.32 (0.08)**^§^** | 1.13 (0.19) | 1.12 (0.10) | - | - | - | 1.22 (0.12)* | 1.36 (0.14)**^§^** | 1.30 (0.08)**^§^** |
| Ucp3 | 1.60 (0.17)**^§^** | 0.98 (0.21) | 0.94 (0.09) | - | - | - | 1.46 (0.09)**^§^** | 1.41 (0.13)**^#^** | 1.19 (0.09) |
| Cox4i1 | 1.21 (0.11)**^§^** | 1.25 (0.04)**^§^** | 1.39 (0.06)**^§^** | 1.13 (0.05) | 1.08 (0.06) | 1.44 (0.08)**^§^** | 1.02 (0.04) | 1.47 (0.05)**^§^** | 1.45 (0.04)**^§^** |
| **Secreted Factors** | | | | | | | | | |
| Adipoq | 1.22 (0.15) | 1.45 (0.12)**^§^** | 1.15 (0.12) | - | - | - | 1.44 (0.12) | 1.69 (0.07) | 2.15 (0.21)**^§^** |
| Mstn | 1.60 (0.13)**^§^** | 1.98 (0.12) **^§^** | 1.70 (0.14) **^§^** | - | - | - | - | - | - |
| Fgf21 | - | - | - | 1.56 (0.32) | 1.70 (0.66) | 1.82 (0.54)* | - | - | - |

**^A^**Fold change indicates the ratio of relative quantification in GW501516-, PF-879- or GW501516 plus PF-879-treated mice over that of vehicle treated mice (i.e., 2^− ΔΔCT^). (n = 8/group). The fold changes marked with *****, **^#^** and **^§^** are significantly differentially expressed genes in treated groups compared to vehicle of p < 0.05, 0.01 and 0.001, respectively. Gene expression levels below the limit of detection are noted with a dash (-).

**^B^**Genes: The gene symbols, official names, and reference sequence identifiers are detailed in **Table S2.**
