# Supplementary material for: Postnatal PPARδ Activation and Myostatin Inhibition Exert Distinct yet Complimentary Effects on the Metabolic Profile of Obese Insulin-Resistant Mice
Source: PLoS One. 2010 Jun 25;5(6):e11307. doi: 10.1371/journal.pone.0011307 (PMC2892469; doi:10.1371/journal.pone.0011307)
Supplement: Table S2 — Symbols, aliases, and official names of genes examined by PCR array. (0.08 MB DOC) [file pone.0011307.s004.doc]

|  |  | | |
| --- | --- | --- | --- |
| **Gene Symbol** | **Alias**  **Used** | **Official Gene Name** | **Ref Seq #** |
| **Insulin Signaling & Glucose Metabolism** | | | |
| Irs1 |  | Insulin receptor substrate 1 | NM_010570 |
| Irs2 |  | Insulin receptor substrate 2 | NM_001081212 |
| Slc2a1 | Glut1 | Solute carrier family 2 (facilitated glucose transporter), member 1 | NM_011400 |
| Slc2a2 | Glut2 | Solute carrier family 2 (facilitated glucose transporter), member 2 | NM_031197 |
| Slc2a3 | Glut3 | Solute carrier family 2 (facilitated glucose transporter), member 3 | NM_011401 |
| Slc2a4 | Glut4 | Solute carrier family 2 (facilitated glucose transporter), member 4 | NM_009204 |
| Gck | Gk | Glucokinase | NM_010292 |
| Hk2 |  | Hexokinase 2 | NM_013820 |
| Pfkm |  | Phosphofructokinase, muscle | NM_021514 |
| Pfkl |  | Phosphofructokinase, liver, B-type | NM_008826 |
| Pdk4 |  | Pyruvate dehydrogenase kinase, isoenzyme 4 | NM_013743 |
| Pepck | Pck1 | Phosphoenolpyruvate carboxykinase 1, cytosolic | NM_011044 |
| G6Pc |  | Glucose-6-phosphatase, catalytic | NM_008061 |
| Fbp2 |  | Fructose bisphosphatase 2 | NM_007994 |
| **Fatty Acid Metabolism** | | | |
| Cd36 |  | CD36 antigen | NM_007643 |
| Slc27a1 |  | Solute carrier family 27 (fatty acid transporter), member 1 | NM_011977 |
| Fabp3 |  | Fatty acid binding protein 3, muscle and heart | NM_010174 |
| Acaca | Acc1 | Acetyl-Coenzyme A carboxylase alpha | NM_133360 |
| Acacb | Acc2 | Acetyl-Coenzyme A carboxylase beta | NM_133904 |
| Acadm |  | Acyl-Coenzyme A dehydrogenase, medium chain | NM_007382 |
| Cpt1a |  | Carnitine palmitoyltransferase 1a, liver | NM_013495 |
| Cpt1b |  | Carnitine palmitoyltransferase 1b, muscle | NM_009948 |
| Gpam | Gpat1 | Glycerol-3-phosphate acyltransferase, mitochondrial | NM_008149 |
| Dgat1 |  | Diacylglycerol O-acyltransferase 1 | NM_010046 |
| Dgat2 |  | Diacylglycerol O-acyltransferase 2 | NM_026384 |
| Fasn | - | Fatty acid synthase | NM_007988 |
| Mlxipl | Chrebp | MLX interacting protein-like | NM_021455 |
| Srebf1 | Srebp1 | Sterol regulatory element binding transcription factor 1 | NM_011480 |
| Nr1h3 | Lxrα | Nuclear receptor subfamily 1, group H, member 3 | NM_013839 |
| Pklr |  | Pyruvate kinase liver and red blood cell | NM_013631 |

|  |  | | |
| --- | --- | --- | --- |
| **Gene Symbol** | **Alias**  **Used** | **Official Gene Name** | **Ref Seq #** |
| **Mitochondrial Biogenesis and Oxidative Metabolism** | | | |
| Ppargc1a | Pgc1α | Peroxisome proliferative activated receptor, gamma, coactivator 1 alpha | NM_008904 |
| Ppargc1b | Pgc1β | Peroxisome proliferative activated receptor, gamma, coactivator 1 beta | NM_133249 |
| Ppara | Pparα | Peroxisome proliferator activated receptor alpha | NM_011144 |
| Ppard | Pparδ | Peroxisome proliferator activator receptor delta | NM_011145 |
| Esrra |  | Estrogen related receptor, alpha | NM_007953 |
| Mfn2 |  | Mitofusin 2 | NM_133201 |
| Ucp1 |  | Uncoupling protein 1 (mitochondrial, proton carrier) | NM_009463 |
| Ucp2 |  | Uncoupling protein 2 (mitochondrial, proton carrier) | NM_011671 |
| Ucp3 |  | Uncoupling protein 3 (mitochondrial, proton carrier) | NM_009464 |
| Cox4i1 |  | Cytochrome c oxidase subunit IV isoform 1 | NM_009941 |
| **Secreted Factors** | | | |
| Adipoq |  | Adiponectin, C1Q and collagen domain containing | NM_009605 |
| Mstn |  | Myostatin | NM_010834 |
| Fgf21 |  | Fibroblast growth factor 21 | NM_020013 |
